# Synthesis and Characterization of a π‑Extended Clar’s Goblet

**DOI:** 10.1021/jacs.5c07588

**Published:** 2025-10-20

**Authors:** Shantanu Mishra, Manuel Vilas-Varela, Igor Rončević, Fabian Paschke, Florian Albrecht, Leo Gross, Diego Peña

**Affiliations:** † Department of Physics, Chalmers University of Technology, 412 96 Gothenburg, Sweden; ‡ 54174 IBM Research Europe − Zurich, 8803 Rüschlikon, Switzerland; § Center for Research in Biological Chemistry and Molecular Materials (CiQUS) and Department of Organic Chemistry, 16780 University of Santiago de Compostela, 15782 Santiago de Compostela, Spain; ∥ Department of Chemistry, 5292 University of Manchester, Oxford Road, Manchester M13 9PL, United Kingdom; ⊥ Oportunius, Galician Innovation Agency (GAIN), 15702 Santiago de Compostela, Spain

## Abstract

Concealed non-Kekulé polybenzenoid hydrocarbons have no sublattice imbalance yet cannot be assigned a classical Kekulé structure, leading to an open-shell ground state with potential applications in organic spintronics. They constitute an exceedingly small fraction of the total number of polybenzenoid hydrocarbons that can be constructed for a given number of benzenoid rings, and their synthesis remains challenging. The archetype of such a system is the Clar’s goblet (C_38_H_18_), a diradical proposed by Erich Clar in 1972 and recently synthesized on a Au(111) surface. Here, we report the synthesis of a π-extended Clar’s goblet (C_76_H_26_), a tetraradical concealed non-Kekulé polybenzenoid hydrocarbon, by a combined in-solution and on-surface synthetic approach. By low-temperature scanning tunneling microscopy and atomic force microscopy, we characterized individual molecules adsorbed on a Cu(111) surface. We provide insights into the electronic properties of this elusive molecule, including the many-body nature of its ground and excited states, by mean-field and multiconfigurational quantum chemistry calculations.

## Graphical abstract

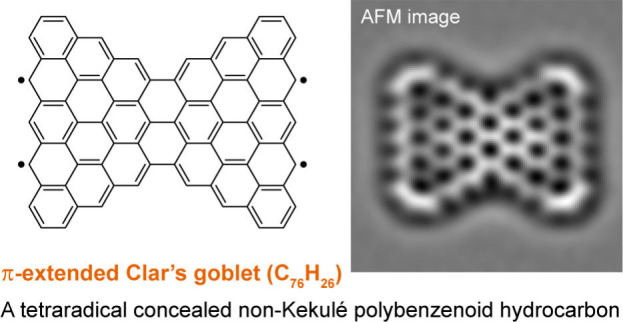

Polybenzenoid hydrocarbons (PBHs) may be broadly distinguished on the basis of the presence or absence of Kekulé valence structures, with important implications for their chemical and electronic properties, such as reactivity, aromaticity, and magnetism.^1–3^ Emergence of magnetism in PBHs may be understood from the interplay of two competing phenomena, namely, (a) the intramolecular hybridization, which drives a closed-shell electronic structure consisting of bonding and antibonding π-orbital pairs in the electronic energy spectrum, such as the highest occupied and lowest unoccupied molecular orbitals (HOMO and LUMO), and (b) the Coulomb repulsion that penalizes double occupation of an orbital and drives an open-shell electronic structure with singly occupied molecular orbitals (SOMOs). In this simple picture, Kekulé PBHs exhibit a finite hybridization-induced gap between the frontier molecular orbitals, and therefore, a critical value of Coulomb repulsion or system size (that governs the hybridization-induced gap for a homologous series of PBHs) is required for the open-shell solution to become the ground state. In contrast, in non-Kekulé PBHs, it is impossible to pair all *p*_*z*_ electrons into π bonds, and such molecules always contain unpaired electrons. A typical example of non-Kekulé PBHs is the family of [*n*]­triangulenes (Figure 1a), which are triangular PBHs containing *n* benzenoid rings along each edge. In non-Kekulé PBHs such as [*n*]­triangulenes, the hybridization-induced gap between the frontier molecular orbitals is negligible, and the inclusion of an arbitrarily small Coulomb repulsion triggers spin polarization, resulting in an open-shell ground state. The underlying reason for the non-Kekulé structure of [*n*]­triangulenes is an inherent sublattice imbalance in the bipartite honeycomb lattice. [*n*]­Triangulenes have a sublattice imbalance of *n* – 1, and therefore a ground state total spin quantum number of (*n* – 1)/2 from Ovchinnikov’s rule.^4,5^ In the literature, such molecules have been referred to as *obvious* non-Kekulé PBHs,^6^ and many of them have recently been synthesized both in solution^7–9^ and on surfaces.^10–19^ However, sublattice imbalance is not a necessary condition to generate non-Kekulé PBHs, and there are examples of so-called *concealed* non-Kekulé PBHs^6^ that cannot be assigned a classical Kekulé structure despite the absence of sublattice imbalance. Ovchinnikov’s rule predicts a singlet ground state for concealed non-Kekulé PBHs. The first such system was proposed by Clar in 1972, the eponymous Clar’s goblet (C_38_H_18_, Figure 1a).^20,21^ In 1974, Gutman showed that concealed non-Kekulé PBHs can be constructed only for *h* ≥ 11, where *h* denotes the number of benzenoid rings in a PBH.^22^ It was further shown that concealed non-Kekulé PBHs constitute a small fraction of the total number of PBHs that can be constructed for a given *h*,^23^ with abundances <0.1% for *h* ≤ 14. To the best of our knowledge, only three concealed non-Kekulé PBHs have been synthesized to date, namely, Clar’s goblet,^24,25^ a [3]­triangulene-dibenzocoronene fused system,^26^ and a [2]­triangulene-perylene fused system.^27^ The rarity of concealed non-Kekulé PBHs, along with the proposed application of these systems as components of spintronic devices,^28–30^ makes them interesting synthetic targets. Here, we report a combined in-solution and on-surface synthesis of a π-extended Clar’s goblet (C_76_H_26_, **ECG**; Figure 1b) on Cu(111) and its characterization by low-temperature scanning tunneling and atomic force microscopies (STM and AFM), along with mean-field and multiconfigurational quantum chemistry calculations.

**Figure 1:**
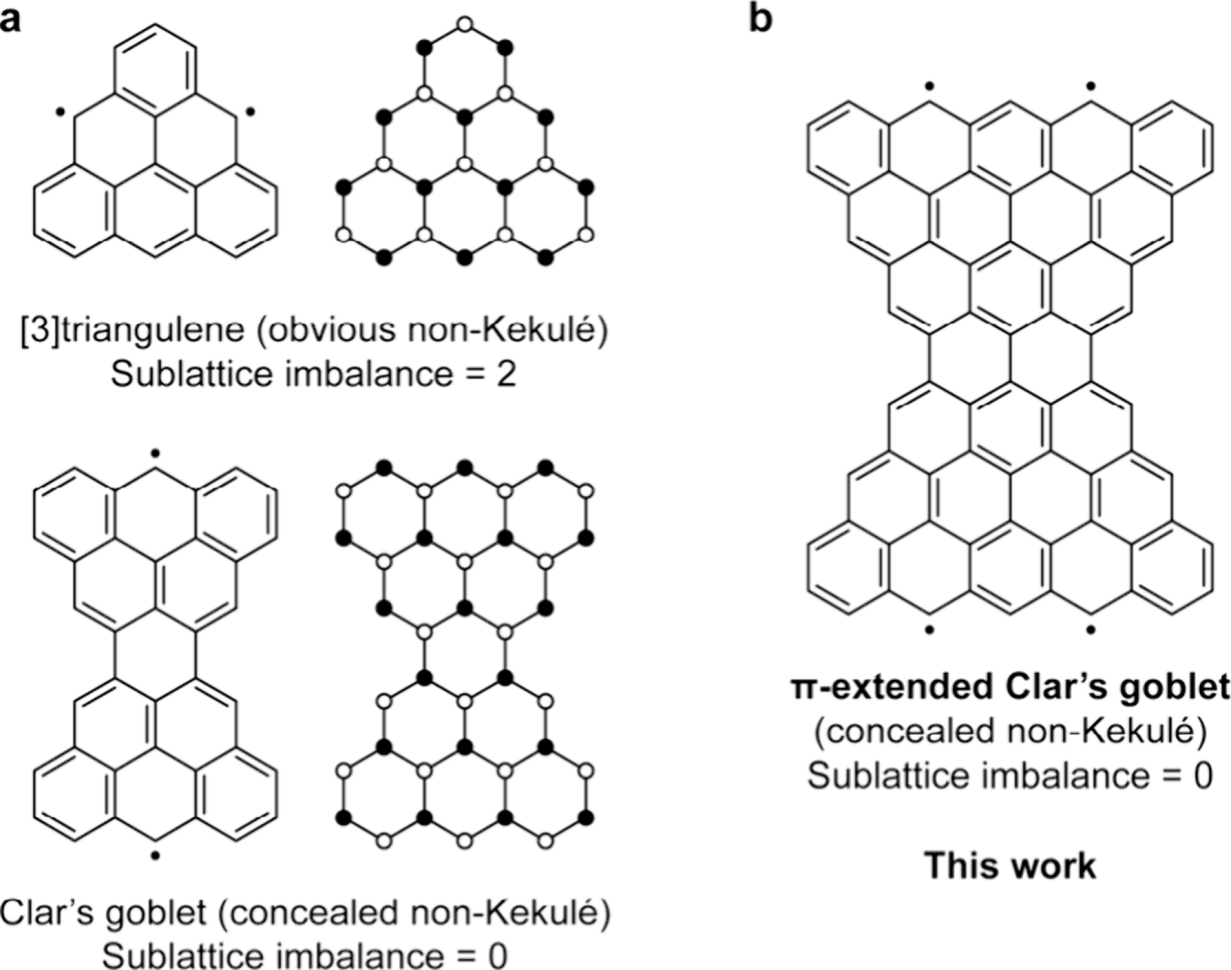
(a) Chemical structures and sublattice representations of [3]­triangulene and Clar’s goblet. Filled and empty circles denote the two sublattices. (b) Chemical structure of the π-extended Clar’s goblet.

The synthesis of **ECG** is based on the on-surface cyclization reactions of compound **1** (Scheme 1). Compound **1** was obtained by solution-phase chemistry in eight steps starting from bianthrone **2**. First, the addition of two equivalents of the organolithium derivative **3**, followed by reduction, led to the formation of the substituted bianthracene **4** in 76% yield. Then, four-fold bromination with *N*-bromosuccinimide (NBS), followed by treatment with sodium acetate (NaOAc), afforded **5** in 33% yield. Ester hydrolysis of **5** under basic conditions, followed by oxidation with pyridinium chlorochromate (PCC), led to the formation of tetra-aldehyde **6** in 49% yield. Finally, addition of four equivalents of **3**, followed by BF_3_-promoted 4-fold intramolecular Friedel–Crafts reaction, led to the isolation of **1** as a mixture of diastereoisomers in 70% yield. Details on solution synthesis and characterization are reported in Figures S1–S12.

**Scheme 1:**
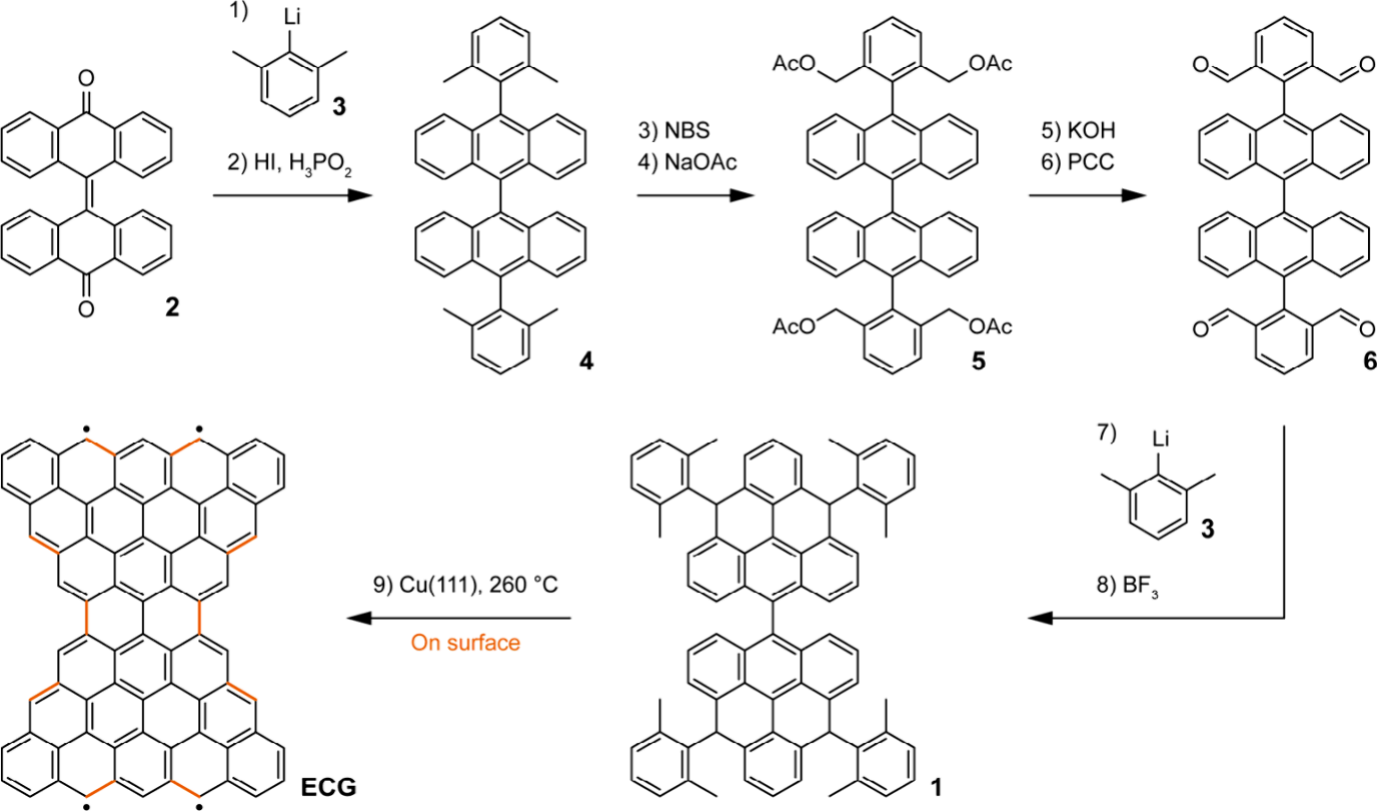
Synthetic Route toward ECG^a^

a Highlighted bonds in **ECG** are formed from on-surface reactions of **1**.

We first attempted the on-surface synthesis of **ECG** on Au(111), which is the least reactive of all coinage metal surfaces and where many open-shell PBHs are shown to be weakly physisorbed without considerable alteration of their gas-phase electronic structure. A submonolayer coverage of **1** was deposited on a Au(111) surface held at 10 K, and the surface was annealed to 600 K for 5 minutes to promote the on-surface reactions. Following this protocol, we observed a universal loss of methyl groups from **1**, which led to the formation of pentagonal rings upon cyclodehydrogenation reactions (Figure S13). Other undesired reactions on Au(111) included the loss of one or more xylyl groups, precursor fragmentation, and intermolecular dehydrogenative cross-coupling reactions (Figure S14). We did not find **ECG** from the STM imaging of more than 100 isolated molecules on Au(111). We then attempted the synthesis of **ECG** on Cu(111), with the rationale that the higher catalytic activity of Cu(111) compared with Au(111) would require lower temperatures to trigger cyclization reactions, potentially avoiding the problem of methyl cleavage. Moreover, the higher diffusion barrier of molecules on Cu compared to Au could also reduce intermolecular reactions, as observed in previous studies.^13,14,31^ Figure 2a presents an overview STM image after annealing a submonolayer coverage of **1** on Cu(111) at 530 K for 5 minutes, wherein the surface was covered by mostly isolated molecules. Approximately 25% of the molecules on the surface corresponded to **ECG**. Figure 2b,c presents AFM images of **ECG** on Cu(111), showing the expected atomic structure of the molecule. In Figure S15, we present AFM images of molecules on Cu(111) that do not correspond to **ECG**, resulting from loss or migration of methyl or xylyl groups, incorporation of carbon atoms, or precursor fragmentation. In AFM imaging (Figures 2b and S16), the central pyrene moiety of **ECG** is imaged brighter (that is, with a more positive frequency shift Δ*f*) because of stronger repulsive forces, whereas the benzenoid rings along the long zigzag edges appear darker. This may indicate a nonplanar adsorption conformation of **ECG**, wherein the carbon atoms at the zigzag edges (that harbor the highest spin density as shown in Figure 3) strongly interact with the underlying Cu atoms and are pulled toward the surface. Density functional theory calculations of **ECG** on Cu(111) confirm this scenario (Figure S17), with Hirschfeld charge analysis indicating substantial electron transfer from Cu(111) to **ECG** (Figure S18). Our observations are in line with a previous study of [7]­triangulene on Cu(111) where the molecule was found to chemisorb on the Cu surface with a nonplanar adsorption geometry.^14^

**Figure 2:**
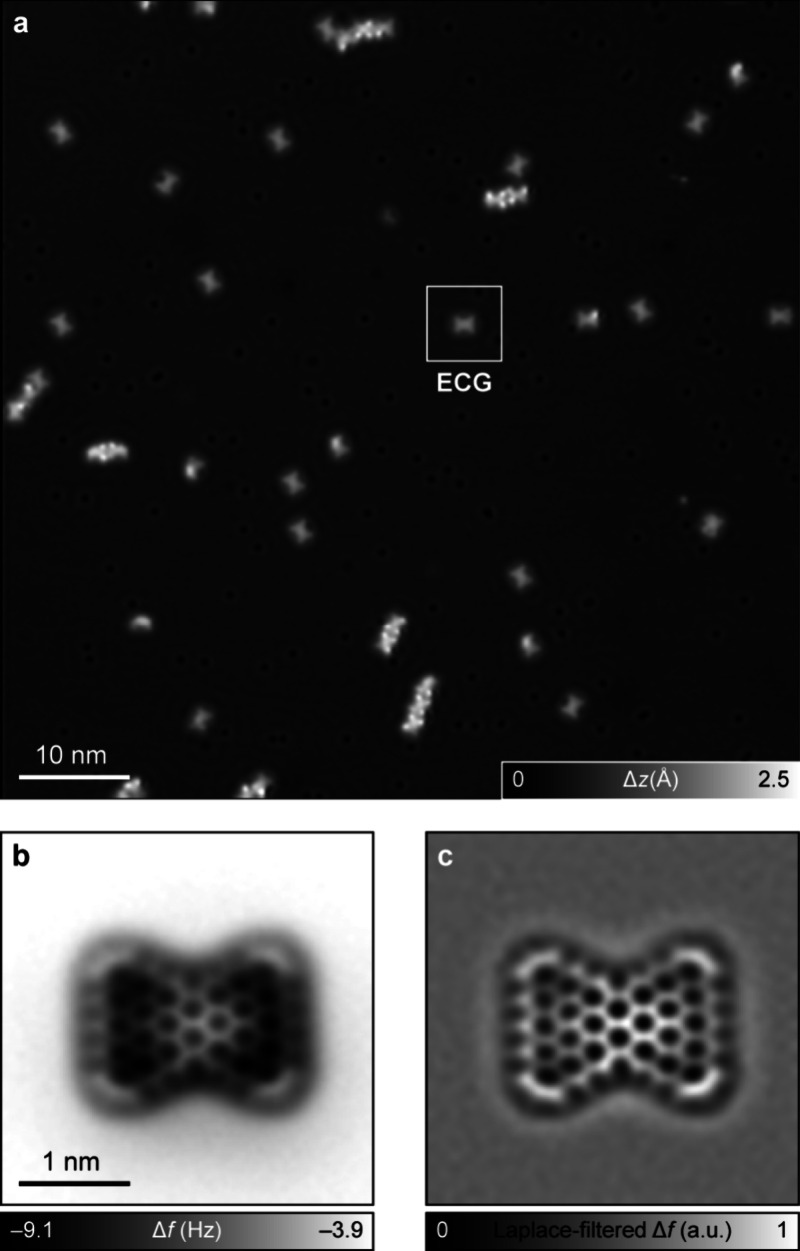
On-surface synthesis and structural characterization of **ECG**. (a) Overview STM image after annealing **1** on Cu(111). Scanning parameters: bias voltage *V* = 0.2 V, tunneling current *I* = 0.5 pA. Δ*z* denotes the tip height. (b) AFM image of the highlighted **ECG** molecule in (a). STM set-point: *V* = 0.2 V, *I* = 0.5 pA on Cu(111); Δ*z* = −2.8 Å. (c) Corresponding Laplace-filtered AFM image. a.u. denotes arbitrary units.

**Figure 3:**
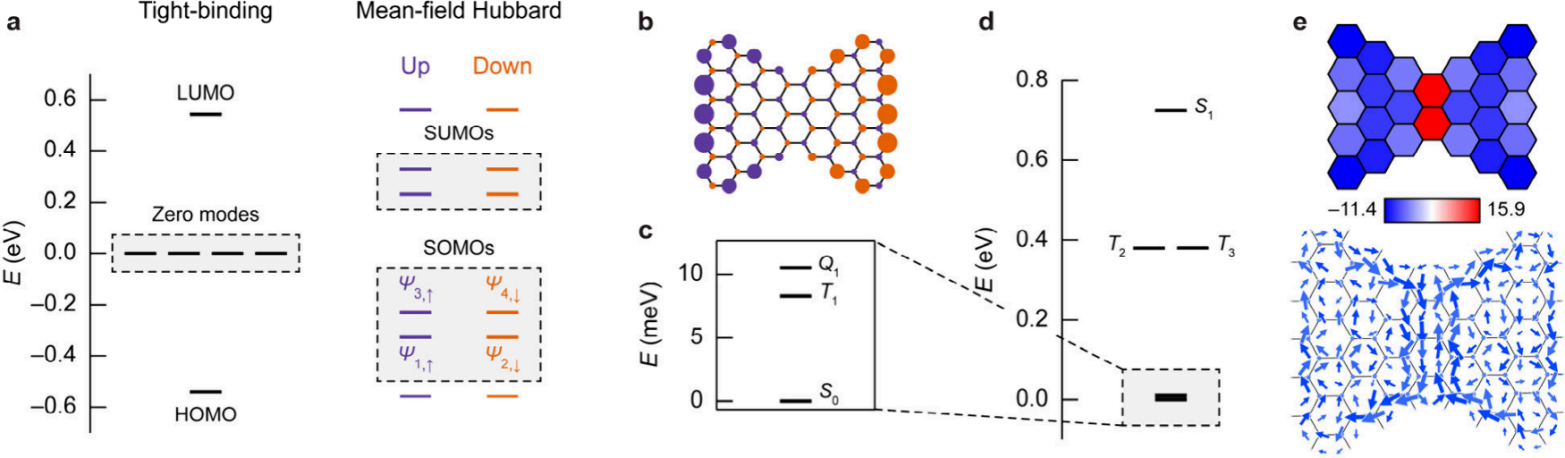
Theoretical electronic characterization of **ECG**. (a) Nearest-neighbor tight-binding (left) and MFH (right) spectra of **ECG**. (b) MFH spin polarization plot of **ECG**, expressed as the difference in the mean populations of spin up and spin down electrons. Size and color of the circles denote the value (the largest and smallest absolute values are 0.295 and 0.017 electrons) and the sign of spin polarization, respectively. (c, d) CASSCF­(4,4)-DDCI spectrum of **ECG**. The low-energy manifold of spin states is shown in (c). The labels *S*, *T*, and *Q* denote singlet, triplet, and quintet states, respectively, and 0, 1, and 2 denote the energetic order (low to high, respectively) of the states of a given multiplicity. (e) NICS(1)_*zz*_ (top) and induced current (bottom) maps of **ECG** in the quintet state. Negative (positive) NICS(1)_*zz*_ values indicate local aromaticity (antiaromaticity).

We now discuss the electronic properties of **ECG** by both mean-field and multiconfigurational calculations. We start by performing nearest-neighbor tight-binding calculations, considering only the *p*_*z*_ orbitals at the carbon sites, which provides an intuitive (albeit overly simplistic) picture of the electronic structure of **ECG**. Figure 3a shows the tight-binding energy spectrum of **ECG**, where the important feature is the presence of four states at zero energy (zero modes) that are populated by four electrons. Away from the zero modes, one finds a series of bonding and antibonding orbital pairs (the first pair of such pairs is indicated as HOMO and LUMO). We then included electronic correlations in **ECG** via the mean-field Hubbard (MFH) approximation, where an intra-atomic Coulomb repulsion term is added to the tight-binding Hamiltonian to account for the energy cost of having a molecular orbital doubly occupied. The MFH solution predicted an open-shell singlet ground state of **ECG**, in agreement with Ovchinnikov’s rule and previous studies.^28,30^ Figure 3a,b shows the MFH spectrum and spin polarization plot of **ECG** in the open-shell singlet state. The degeneracy of the zero modes is now lifted by spin polarization, resulting in the formation of four SOMOs (and the corresponding unoccupied molecular orbitals, SUMOs) labeled as ψ_1_–ψ_4_. The SOMOs are sublattice polarized, with SOMOs of opposite spins localized on different sublattices and on opposite halves of **ECG**.^24,28^ To obtain more accurate insights into the electronic structure of **ECG**, we performed calculations accounting for the multiconfigurational nature of the ground and excited states (see Supporting Information).^32^ Briefly, we performed complete active space self-consistent field (CASSCF) calculations wherein the occupation of the orbitals corresponding to the four zero modes is allowed to vary, while the occupations of the orbitals lower or higher in energy are frozen. The active space thus consists of four electrons in the four zero modes, that is, CASSCF­(4,4). The CASSCF­(4,4) calculations confirmed the open-shell singlet ground state of **ECG**. To accurately determine excited-state energies and exchange interactions, we employed the difference-dedicated configuration interaction (DDCI) method.^33^ DDCI improves upon CASSCF by building a CI space that includes all single and double excitations involving at least one active space electron or orbital. Figure 3c,d shows the CASSCF­(4,4)-DDCI-calculated spectrum of the ground and excited states of **ECG** (see also Table S1). Relative to the (open-shell) singlet ground state, the first excited state is a triplet that is 8 meV higher in energy, followed by the second excited state at 10 meV, which is a quintet. Further up in energy are two nearly degenerate triplet states at 381 meV and a singlet state at 725 meV. Most of these states exhibit a strong multiconfigurational character that cannot be captured by mean-field theories (Figure S19). We also rationalized the many-body states shown in Figure 3c,d in terms of model spin Hamiltonians with linear and nonlinear exchanges and derived the relevant exchange interactions (Figure S20). Finally, we analyzed the aromaticity of **ECG** by calculating induced currents and a nucleus-independent chemical shift (NICS) map in the quintet state (Figures 3e and S21). The induced current map reveals the presence of two diatropic ring currents in the two halves of **ECG**, which meet in the center to produce a strong paratropic current. NICS values confirm aromatic spin-bearing dibenzo­[*bc*,*pq*]­ovalenyl moieties and an antiaromatic central naphthalene moiety, similar to Clar’s goblet^34^ and in agreement with the Clar sextet distribution of **ECG** (Figure 1b).

In summary, by solution-phase and on-surface chemistry, we synthesized a π-extended Clar’s goblet (**ECG**) on Cu(111) and characterized its chemical structure by STM and AFM imaging. DFT calculations indicated the chemisorption of **ECG** on Cu(111). We analyzed the electronic structure of **ECG** through mean-field and multiconfigurational quantum chemistry calculations. We found through these calculations that in the gas phase **ECG** is a tetraradical system with a singlet ground state and close-lying triplet and quintet excited states, which exhibit strong multiconfigurational characters. Our study demonstrates synthetic access to a rare class of polyradical conjugated systems with proposed application in spintronics.

## Supplementary Material



## Notes

Experimental and theoretical methods, details of the solution synthesis of compound **1**, ^1^H and ^13^C NMR spectra, mass spectra, STM and AFM data, and additional calculations (PDF)

## Notes

During the peer review of our work, we became aware of a related study on the synthesis of **ECG** on Au(111).^35^ Our predicted singlet–triplet gap of **ECG** by multiconfigurational calculations is in good agreement with the experimentally measured spin excitation gap of **ECG** on Au(111).
