# Supplementary material for: Synthesis and Characterization of a π‑Extended Clar’s Goblet
Source: J Am Chem Soc. 2025 Oct 20;147(43):39067–71. doi: 10.1021/jacs.5c07588 (PMC12576809; doi:10.1021/jacs.5c07588)
Supplement: Supplementary file 1 [file ja5c07588_si_001.pdf]

Supporting Information

Synthesis and Characterization of a π-Extended Clar's Goblet

Shantanu Mishra,[1,2] Manuel Vilas-Varela,[3] Igor Rončević,[4] Fabian Paschke,[2] Florian Albrecht,[2] Leo Gross,[2] and Diego Peña,[3,5]

[1]Department of Physics, Chalmers University of Technology, 412 96 Gothenburg, Sweden.

[2]IBM Research Europe – Zurich, 8803 Rüschlikon, Switzerland.

[3]Center for Research in Biological Chemistry and Molecular Materials (CiQUS) and Department of Organic Chemistry, University of Santiago de Compostela, 15782 Santiago de Compostela, Spain.

[4]Department of Chemistry, University of Manchester, Oxford Road, Manchester M13 9PL, United Kingdom.

[5]Oportunius, Galician Innovation Agency (GAIN), 15702 Santiago de Compostela, Spain.

Contents

1. Methods Page 2
2. Supporting data Page 14
3. References Page 20

1. Methods

1.1. Solution synthesis and characterization.

Starting materials were purchased reagent grade from TCI and Sigma-Aldrich and used without further purification. Bianthrone **2** was obtained following reported procedures.[1] Organolithium reagent **3** was prepared by treatment of 2-iodo-1,3-dimethylbenzene with *n*-butyllithium. All reactions were carried out in flame-dried glassware under an inert atmosphere of purified Ar using Schlenk techniques. Thin-layer chromatography was performed on Silica Gel 60 F-254 plates (Merck). Column chromatography was performed on silica gel (40-60 μm). Nuclear magnetic resonance (NMR) spectra were recorded on a Bruker Varian Inova 500 spectrometer. Mass spectra, using the atmospheric pressure chemical ionization (APCI) method, were recorded on a Bruker MicroTOF spectrometer. Mass spectrum and high-resolution mass spectrum are denoted as MS and HRMS, respectively.

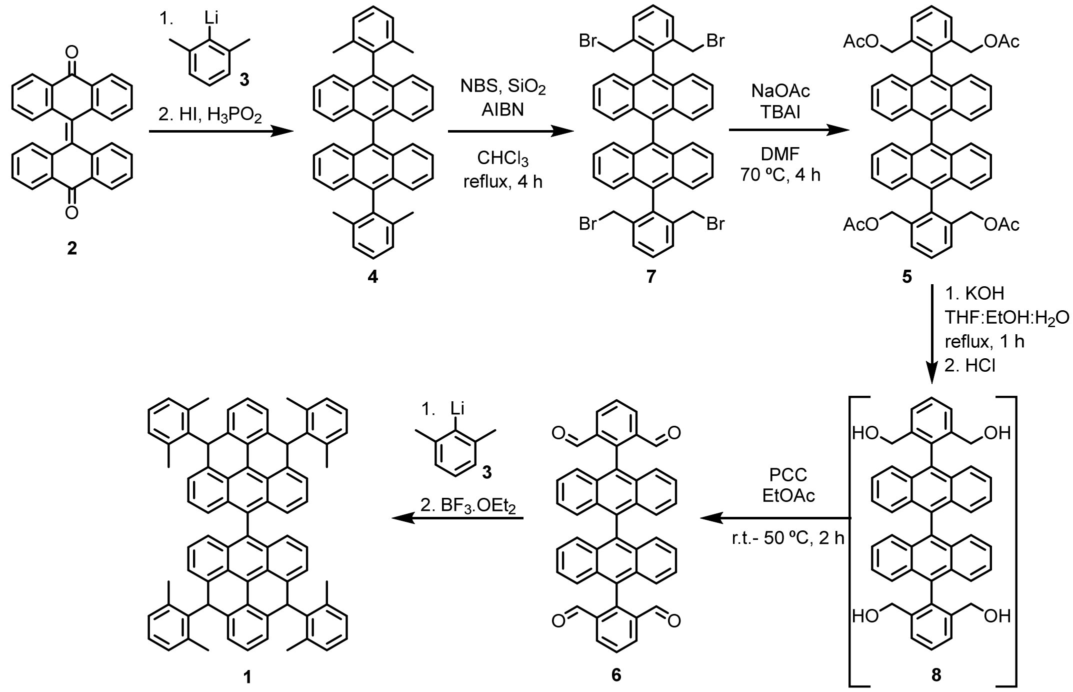

Figure S1. Synthetic route toward **1**.

Synthesis of 10,10'-bis(2,6-dimethylphenyl)-9,9'-bianthracene (4)

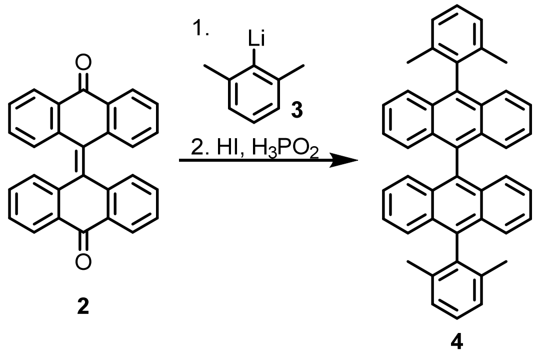

Figure S2. Synthesis of **4**.

Over a solution of **3** (13.0 mmol) in Et_2O (60 mL), compound **2** (1.00 g, 2.60 mmol) was added at 0 ºC. The resulting mixture was allowed to reach room temperature and stirred for 16 h. Then, AcOH (1.00 mL) was added, and the solvent was removed under reduced pressure. The residue was suspended in AcOH (40 mL). Then, H_3PO_2 (20 mL) and HI (4 mL) were added. The resulting suspension was heated at 90 ºC for 3 h. After cooling to room temperature, H_2O (100 mL) was added, and the precipitate was filtered and washed with H_2O (2 × 15 mL) and MeOH (2 × 10 mL). The obtained solid was purified by column chromatography (SiO_2; hexane:CH_2Cl_2 4:1) to afford **4** (1.11 g, 76%) as a yellow solid. **^{1}H-NMR** (500 MHz, $CDCl_3$) δ: 7.64 (d, *J* = 8.7 Hz, 4H), 7.49 – 7.41 (m, 2H), 7.41 – 7.29 (m, 8H), 7.27 – 7.14 (m, 8H), 2.00 (s, 12H) ppm. **^{13}C- NMR** (125 MHz, $CDCl_3$) δ: 138.0 (C), 136.5 (C), 133.2 (C), 131.8 (C), 129.6 (C), 127.9 (CH), 127.7 (CH), 127.4 (CH), 126.4 (CH), 125.8 (CH), 125.7 (CH), 20.5 (CH_3) ppm. **MS (APCI)** *m/z* (%): 562 (M+, 100). **HRMS (APCI)**: $C_{44}H_{35}$; calculated: 563.2733, found: 562.2734.

Synthesis of 10,10'-bis(2,6-bis(bromomethyl)phenyl)-9,9'-bianthracene (7)

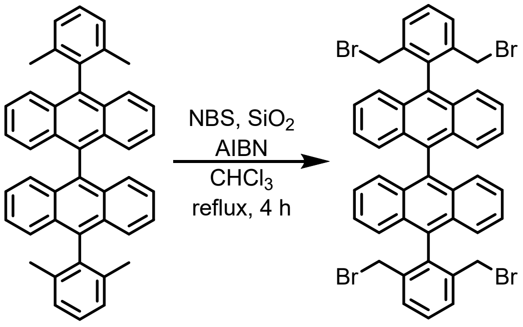

Figure S3. Synthesis of **7**.

Over a mixture of **4** (100 mg, 0.18 mmol), NBS (160 mg, 0.90 mmol) and SiO_2 (150 mg) in $CHCl_3$ (10 mL), a catalytic amount of AIBN was added. The mixture was heated at reflux for 4 h. The solvent was removed under reduced pressure and the residue was purified by column chromatography (SiO_2; hexane:CH_2Cl_2 4:1 to 2:1) to afford **7** (50 mg, 35%) as a yellowish solid. **^{1}H NMR** (500 MHz, $CDCl_3$) δ: 7.81 (d, *J* = 7.7 Hz, 4H), 7.68 (dd, *J* = 8.4, 6.9 Hz, 2H), 7.56 (d, *J* = 8.7 Hz, 4H), 7.37 (ddd, *J* = 8.7, 5.3, 2.4 Hz, 4H), 7.24 (dd, *J* = 5.9, 4.0 Hz, 8H), 4.19 (s, 8H) ppm. **^{13}C NMR** (125 MHz, $CDCl_3$) δ: 138.20 (C), 131.50 (C), 131.30 (CH), 130.30 (C), 129.6 (CH), 127.30 (CH), 126.70 (CH), 126.40 (CH), 126.20 (CH), 31.60 (CH_2). ppm. **MS (APCI)** *m/z* (%): 878 (M+, 100), 799 (18). **HRMS (APCI)**: $C_{44}H_{30}Br_4$; calculated: 873.9076, found: 873.9070.

Synthesis of ([9,9'-bianthracene]-10,10'-diylbis(benzene-2,1,3-triyl))tetrakis(methylene) tetraacetate (5)

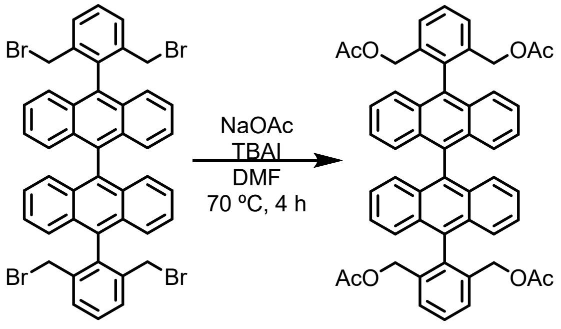

7 **5**

Figure S4. Synthesis of **5**.

A mixture of **7** (50 mg, 0.06 mmol), NaOAc (50 mg, 0.60 mmol) and TBAI (5 mg, 0.01 mmol) in DMF (5 mL) was heated at 80 °C for 4 h. After cooling to room temperature, H_2O (50 mL) was added, and the mixture was extracted with EtOAc (2 × 15 mL). The combined organic extracts were dried over $MgSO_4$, filtered, and evaporated under reduced pressure. The residue was purified by column chromatography (SiO_2; hexane:EtOAc 4:1) to afford **5** (45 mg, 94%) as a yellowish solid. **^{1}H NMR** (500 MHz, $CDCl_3$) δ: 7.70 (q, *J* = 4.9 Hz, 6H), 7.53 (d, *J* = 8.8 Hz, 4H), 7.37 – 7.29 (m, 4H), 7.20 (d, *J* = 3.5 Hz, 8H), 4.78 (s, 8H), 1.50 (S, 12H) ppm. **^{13}C NMR** (125 MHz, $CDCl_3$) δ: 170.2 (CO), 138.8 (C), 136.4 (C), 134.5 (C), 131.9 (C), 131.4 (C), 130.1 (C), 129.8 (CH), 128.7 (CH), 127.4 (CH), 126.3 (CH), 126.0 (CH), 64.7 (CH_2), 20.1(CH_3) ppm. **MS (APCI)** *m/z* (%): 794 (M+, 100), 735 (3). **HRMS (APCI)**: $C_{52}H_{42}O_8$; calculated: 794.2874, found: 794.2873.

Synthesis of 2,2'-([9,9'-bianthracene]-10,10'-diyl)diisophthalaldehyde (6)

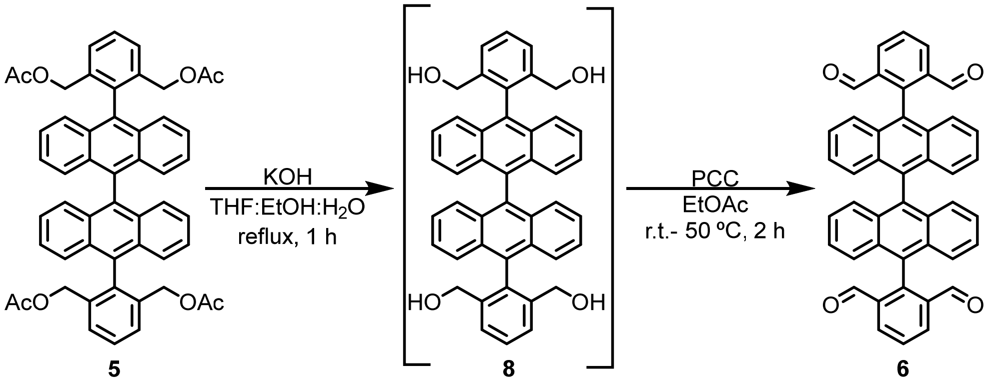

5 **8** **6**

Figure S5. Synthesis of **6**.

A mixture of **5** (510 mg, 0.64 mmol) and KOH (360 mg, 6.40 mmol) in THF:EtOH:H_2O (2:2:2, 20 mL) was refluxed for 1 h. After cooling to room temperature, H_2O (50 mL) was added, and the pH was adjusted to 5 by addition of $HCl_{(aq)}$. The resulting mixture was extracted with EtOAc (5 × 25 mL). The combined organic extracts were dried over $MgSO_4$, filtered and evaporated under reduced pressure. The crude product **8** was suspended in EtOAc:CH_2Cl_2 (3:1, 100 mL), and PCC (690 mg, 3.2 mmol) was portion-wise added at room temperature. After the addition, the mixture was heated at 50 °C for 2 h, cooled to room temperature and quenched by addition of *i*PrOH (5 mL). Solvents were removed under reduced pressure and the residue was purified by column chromatography (SiO_2; CH_2Cl_2) to afford **6** (192 mg, 49%) as a yellowish solid. **^{1}H NMR** (500 MHz, $CDCl_3$) δ: 9.56 (s, 4H), 8.58 (d, *J* = 7.7 Hz, 4H), 7.97 (t, *J* = 7.7 Hz, 2H), 7.53 – 7.40 (m, 8H), 7.33 – 7.20 (m, 8H) ppm. **^{13}C NMR** (125 MHz, $CDCl_3$) δ:

190.7 (CO), 145.8 (C), 136.5 (C), 135.1 (C), 133.3 (CH), 131.9 (C), 130.9 (C), 129.7 (CH), 127.6 (CH), 127.4 (CH), 127.3 (C), 126.8 (CH), 126.2 (CH) ppm. **MS (APCI)** *m/z* (%): 618 (M+, 100). **HRMS (APCI)**: $C_{44}H_{27}O_4$; calculated: 619.1904, found: 619.1881.

Synthesis of 8,8',12,12'-tetrakis(2,6-dimethylphenyl)-8,8',12,12'-tetrahydro-4,4'-bidibenzo[*cd*,*mn*]pyrene (1)

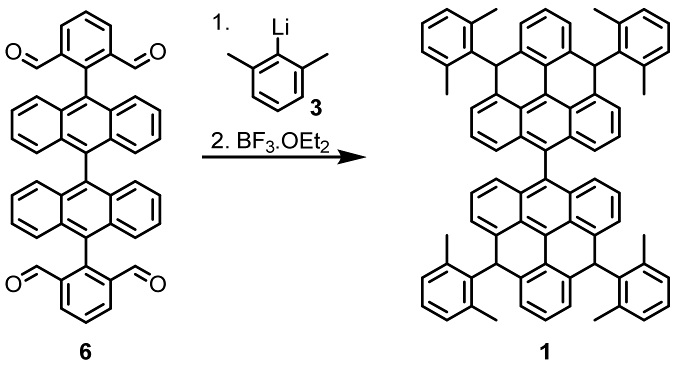

Figure S6. Synthesis of **1**.

Over a solution of **3** (0.20 mmol) in Et_2O (2 mL), a suspension of **6** (12 mg, 0.02 mmol) in Et_2O:THF (1:2, 6 mL) was added, and the resulting mixture was stirred at room temperature for 4 h. Then, H_2O (5 mL) was added, and the mixture was extracted with EtOAc (2 × 10 mL). The combined organic extracts were dried over $MgSO_4$, filtered and evaporated under reduced pressure. The obtained product was dissolved in CH_2Cl_2 (3 mL) and BF_3.OEt_2 (40 μL) was added at 0 ºC. The resulting mixture was allowed to reach room temperature and stirred for 30 min. Then, H_2O (5 mL) was added, the phases were separated, and the aqueous phase was extracted with CH_2Cl_2 (2 × 5 mL). The combined organic extracts were dried over $MgSO_4$, filtered and evaporated under reduced pressure. The residue was purified by column chromatography (SiO_2; hexane: CH_2Cl_2 4:1 to 1:1) to afford **1** (5 mg, 70%) as an orange solid (mixture of diastereomers). **^{1}H NMR** (500 MHz, $CDCl_3$) δ: 7.31 – 7.13 (m, 12H), 7.07 – 6.97 (m, 10H), 6.87 (d, *J* = 6.8 Hz, 4H), 6.80 (d, *J* = 7.6 Hz, 4H), 6.72 – 6.46 (m, 4H), 2.68 (m, 12H), 2.03 – 1.65 (m, 12H) ppm. **MS (APCI)** *m/z* (%): 970 (M+, 100), 865 (8). **HRMS (APCI)**: $C_{76}H_{58}$; calculated: 970.4533, found: 970.4547.

The overall yield for the synthesis of compound **1** was ~8.6%, which is comparable to that of a similar compound previously synthesized by Su et al. (yield ~6%).[2]

^{1}H and ^{13}C NMR spectra

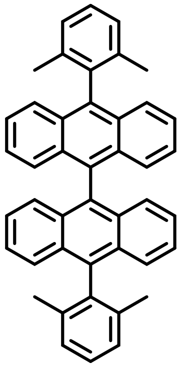

4

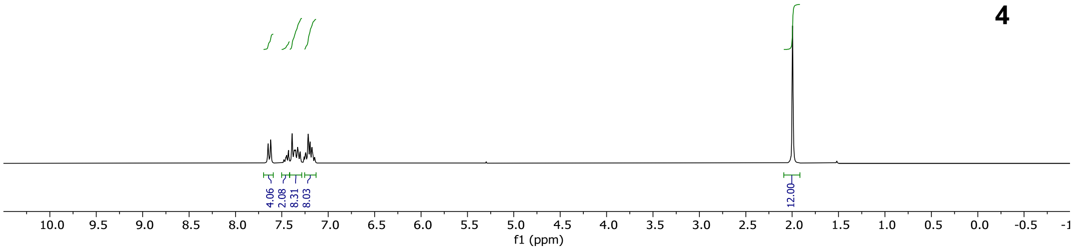

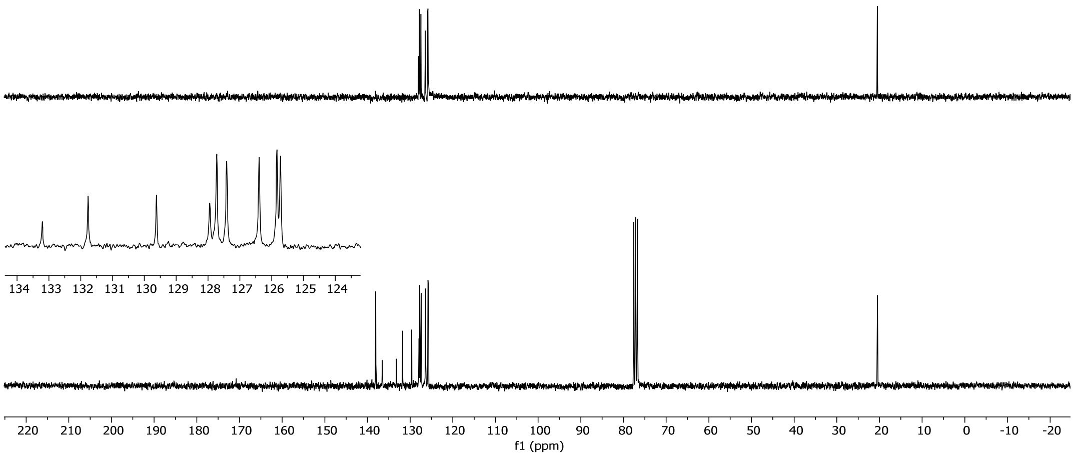

Figure S7. ^{1}H and ^{13}C NMR spectra of **4**.

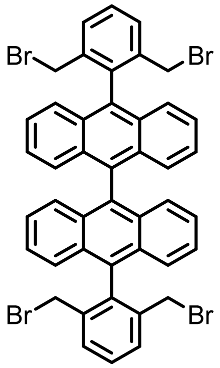

7

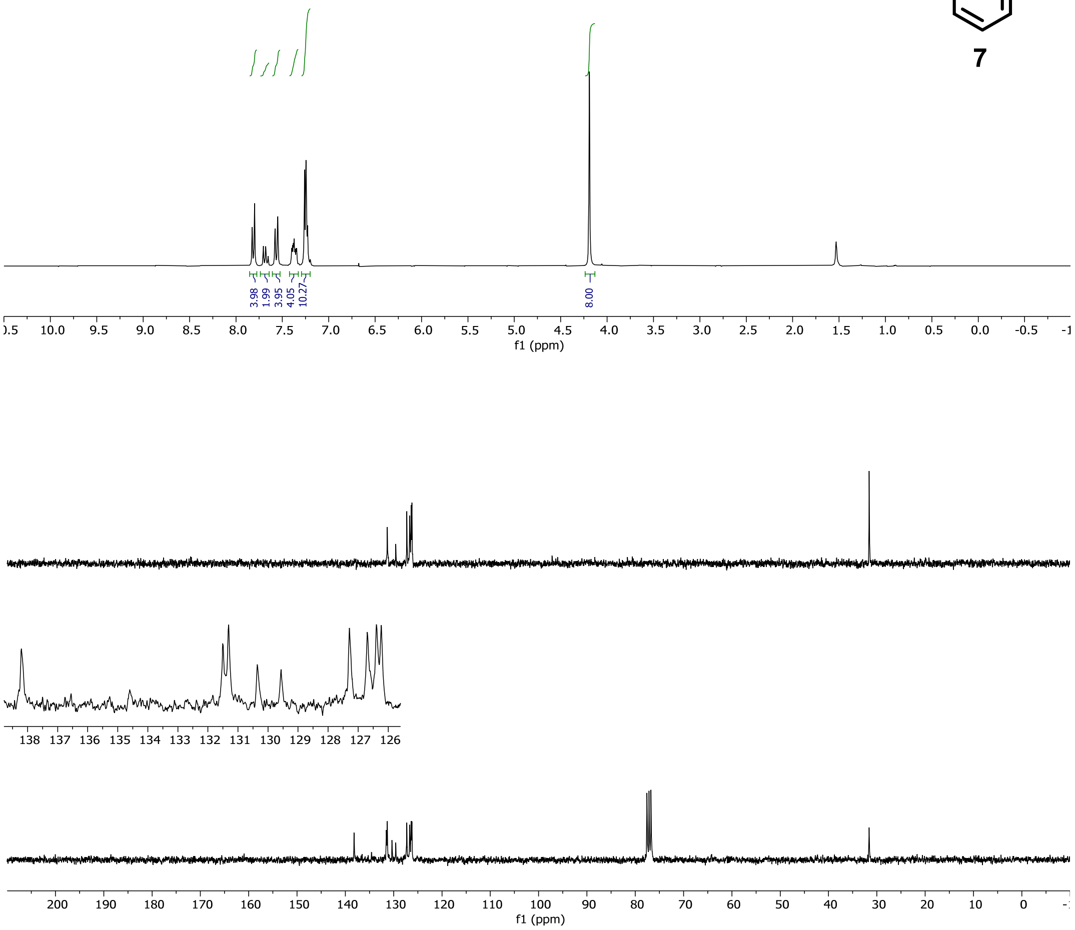

Figure S8. ^{1}H and ^{13}C NMR spectra of **7**.

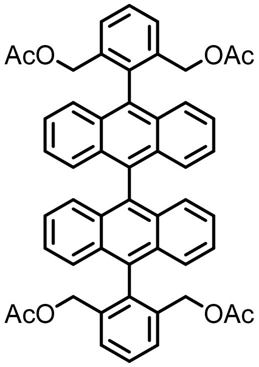

5

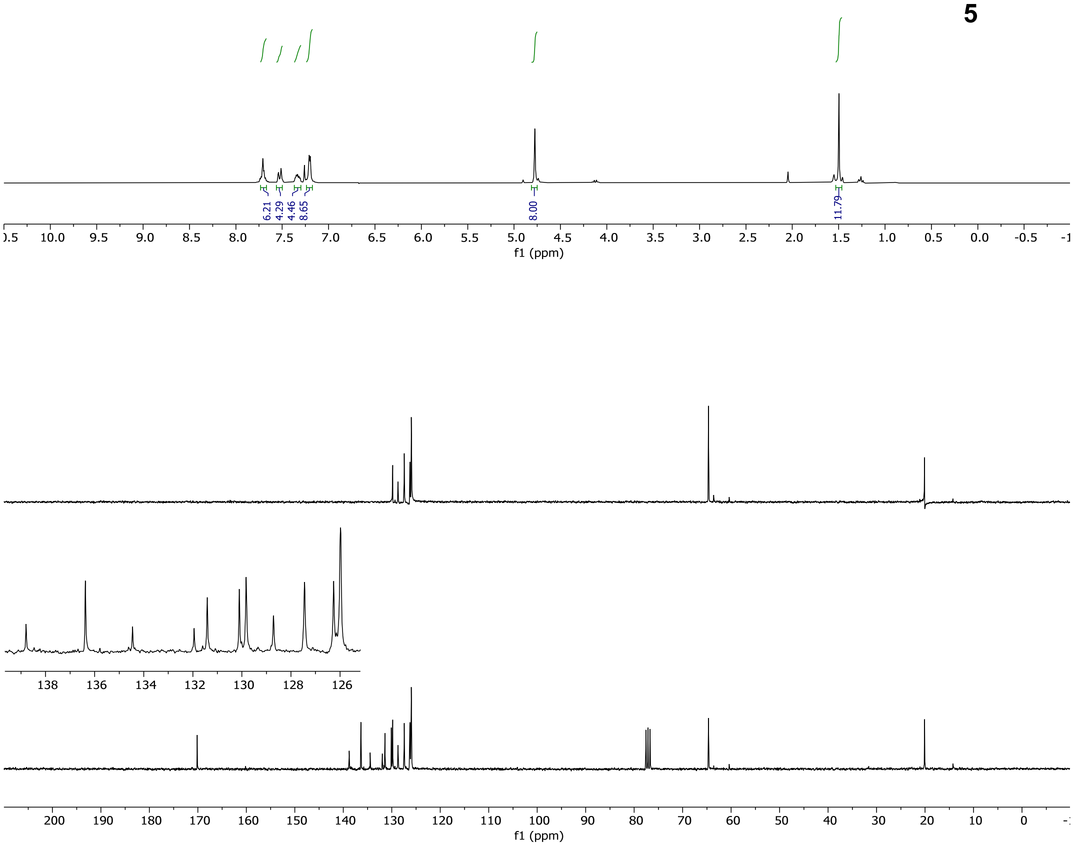

Figure S9. ^{1}H and ^{13}C NMR spectra of **5**.

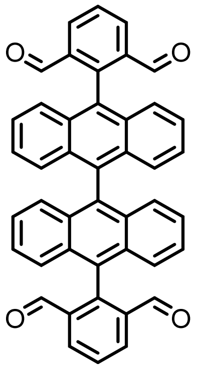

6

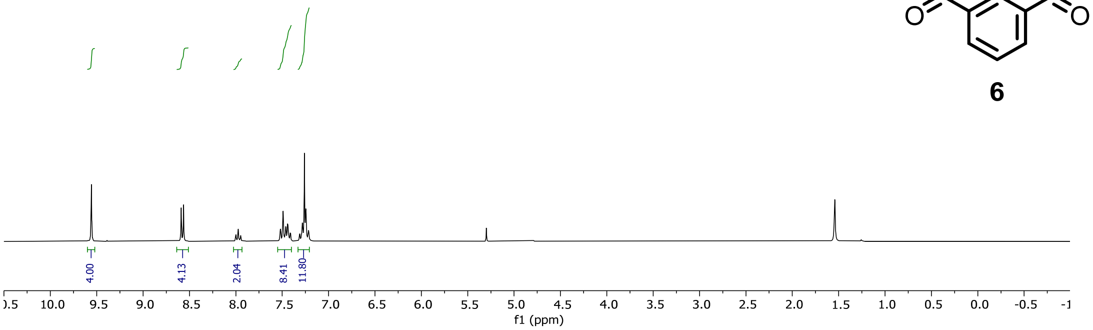

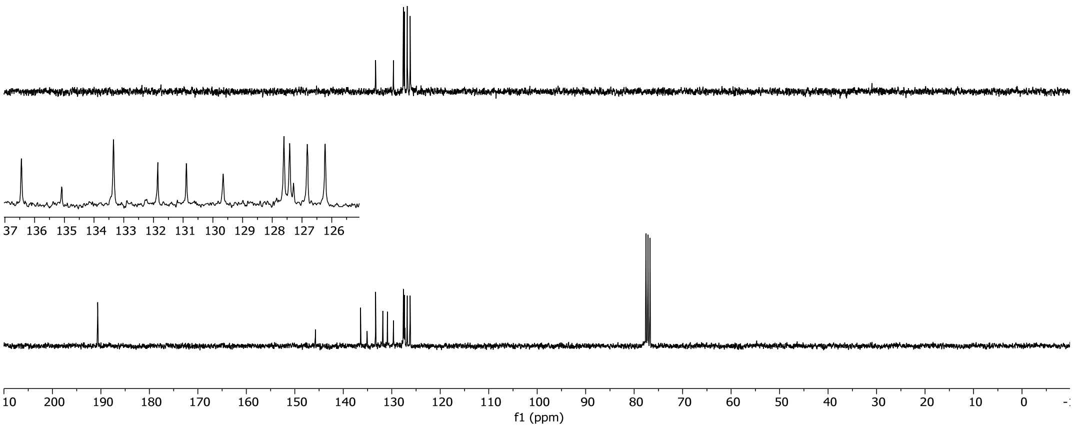

Figure S10. ^{1}H and ^{13}C NMR spectra of **6.**

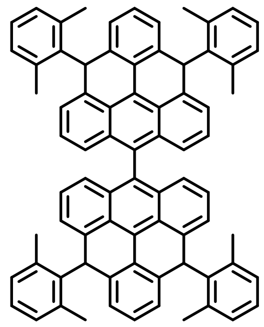

1

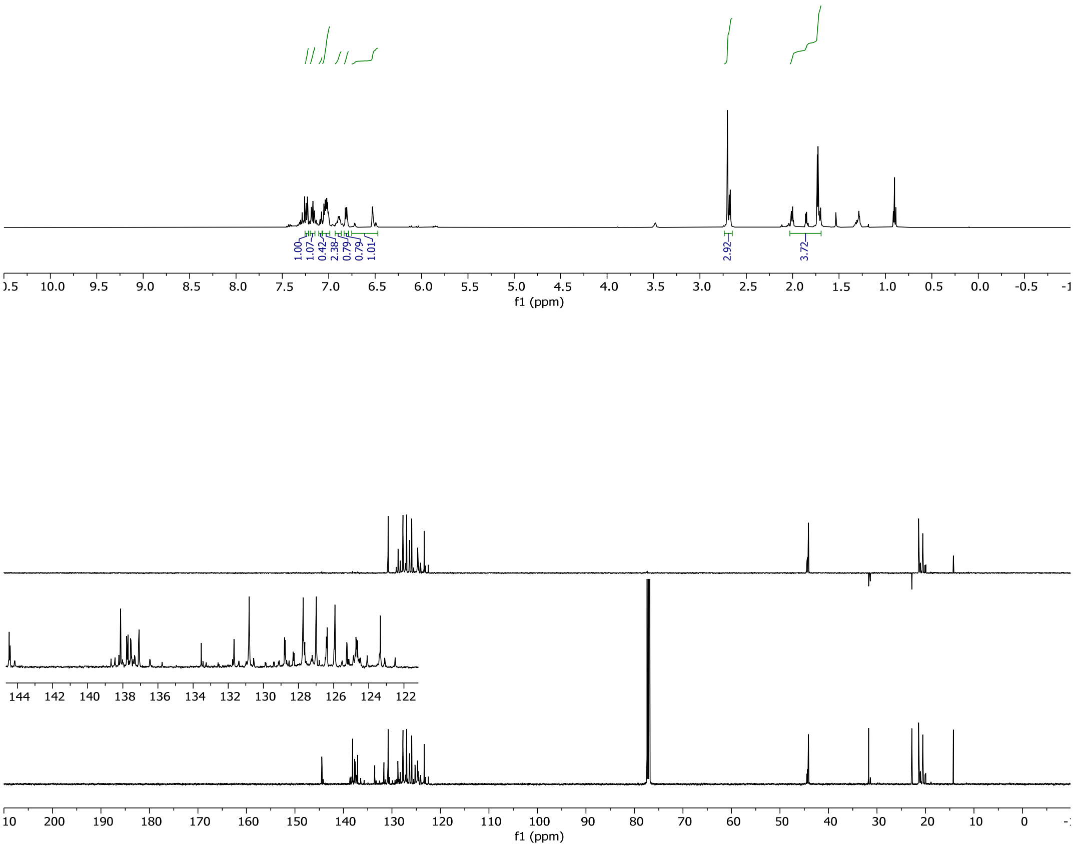

Figure S11. ^{1}H and ^{13}C NMR spectra of **1**.

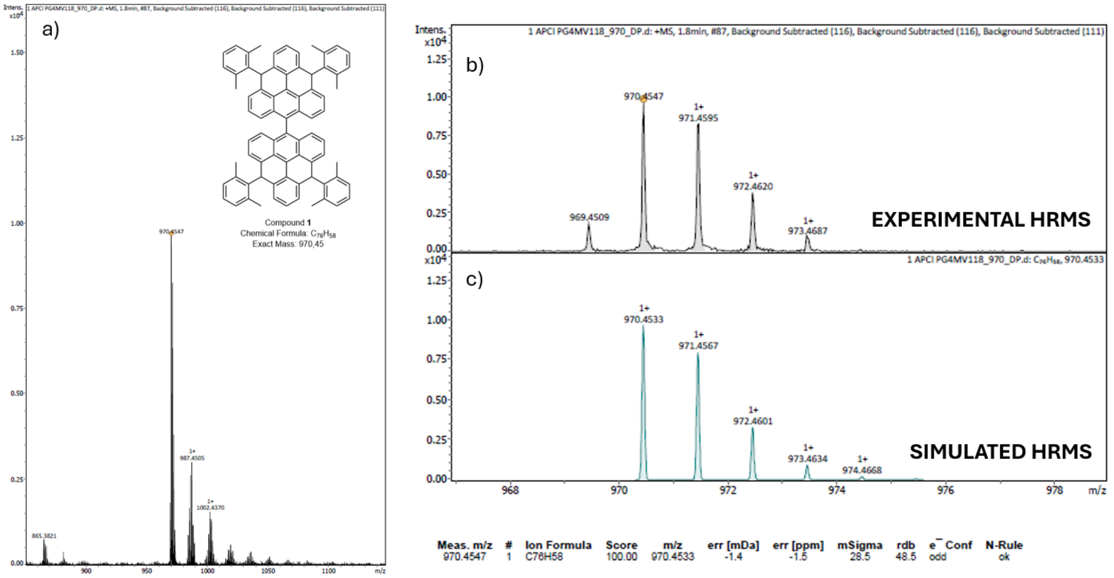

Figure S12. (a) MS (APCI) of **1**, which shows the molecular ion (M^+) at *m/z* = 970 ($C_{76}H_{58}$). The MS also shows peaks at $M^+ + 16$, $M^+ + 32$, $M^+ + 48$, $M^+ + 64$, $M^+ + 80$ and $M^+ + 96$, which are related to the easy oxidation of compound **1** under the MS conditions. (b) HRMS (APCI) of **1**. (c) Simulated HRMS for $C_{76}H_{58}$.

1.2. Sample preparation and scanning probe experiments.

Scanning probe measurements were performed in a home-built STM/AFM setup operating at base pressures below 1×10^{-10} mbar and a base temperature of 5 K. Bias voltages were applied to the sample with respect to the tip. All STM and AFM images were acquired with carbon monoxide-functionalized tips. AFM measurements were performed in non-contact mode with a qPlus[3] sensor. The sensor was operated in frequency modulation mode[4] with a constant oscillation amplitude of 0.5 Å. STM images were acquired in constant-current mode, and AFM images were acquired in constant-height mode with $V = 0$ V. Au(111) and Cu(111) surfaces were cleaned by iterative cycles of sputtering with Ne^+ ions and annealing up to 800 K. NaCl was thermally evaporated on Au(111) and Cu(111) surfaces held at 323 K and 283 K, respectively. This protocol resulted in the growth of predominantly bilayer (100)-terminated NaCl islands, with a minority of third-layer NaCl islands. Submonolayer coverage of **1** on the surfaces was obtained by flashing an oxidized silicon wafer containing the precursor molecules in front of the cold sample in the microscope. Carbon monoxide molecules for tip functionalization were dosed from the gas phase on the cold sample.

1.3. Tight-binding and mean-field Hubbard calculations.

Nearest-neighbor tight-binding and mean-field Hubbard calculations were performed by numerically solving the following Hamiltonian

$$\hat{H} = -t \sum_{\langle i,j\rangle,\sigma} c^{\dagger}_{i,\sigma} c_{j,\sigma} + U \sum_{i,\sigma} \langle n_{i,\sigma}\rangle n_{i,\bar{\sigma}} - U \sum_{i} \langle n_{i,\uparrow}\rangle \langle n_{i,\downarrow}\rangle. \qquad (1)$$

Here, $c^{\dagger}_{i,\sigma}$ and $c_{j,\sigma}$ denote the spin selective ($\sigma \in \{\uparrow, \downarrow\}$ with $\bar{\sigma} \in \{\downarrow, \uparrow\}$) creation and annihilation operator at nearest-neighbor sites i and j, t = 2.7 eV is the nearest-neighbor hopping parameter, U = 4 eV is the on-site Coulomb repulsion, $n_{i,\sigma}$ and $\langle n_{i,\sigma}\rangle$ denote the number operator and mean occupation number at site i, respectively. Orbital electron densities, ρ, of the nth-eigenstate with energy E_n have been simulated from the corresponding state vector $a_{n,i,\sigma}$ by

$$\rho_{n,\sigma}(\vec{r}) = \left|\sum_i a_{n,i,\sigma}\phi_{2p_z}(\vec{r}-\vec{r}_i)\right|^2, \quad (2)$$

where ϕ_{2p_z} denotes the Slater $2p_z$ orbital for carbon.

1.4. Density functional theory calculations.

Both gas-phase and on-surface DFT calculations on **ECG** were performed on the high-spin (quintet) manifold. This choice was made because the quintet state can be described well with a single determinant, that is, the quintet DFT wavefunction has the same symmetry as the molecule. While the open-shell singlet wavefunction can be obtained using broken-symmetry DFT, performing geometry optimizations and evaluating magnetic properties using this wavefunction may yield erroneous results due to unphysical symmetry breaking and spin contamination.

Gas-phase calculations. The **ECG** geometry was optimized at the B3LYP/def2-SVP level of theory[5,6] in the quintet state. Magnetic properties ($NICS(1)_{zz}$ and bond currents) were probed at this geometry and spin state at the B3LYP/def2-TZVP level of theory. These calculations were performed using Gaussian16,[7] with SYSMOIC[8] being employed for bond currents.

On-surface calculations. Calculations were done using VASP at the PBE-D3BJ level of theory.[9,10] A 10×10 supercell of the Cu(111) surface was prepared from a face-centered lattice with the bulk lattice constant (3.614 Å), and 12 Å were added in the *z*-direction. Due to the large size of the supercell, only three layers of Cu atoms were included, and *k*-space was sampled only at the gamma point. The adsorption energy was estimated according to

$$E_{\text{ads}} = E_{\mathbf{ECG@}\text{Cu111}} - E_{\mathbf{ECG}} - E_{\text{Cu111}} \quad (3)$$

The value of $E_{\mathbf{ECG}}$ was estimated by relaxing the high-spin (quintet) geometry of **ECG** in the gas phase, using a sufficiently large unit cell, and the surface energy E_{Cu111} was determined by relaxing the uppermost layer of Cu atoms in the prepared supercell. Finally, $E_{\mathbf{ECG@}\text{Cu111}}$ was obtained by placing **ECG** onto the reconstructed surface in four orientations (with the long axis of **ECG** rotated by ~0°, ~30°, ~60°, and ~90° with respect to the *a* lattice vector) and performing spin-polarized relaxations on the quintet manifold. Relaxations (with forces converged to 0.01 eV/Å) found the two lowest-energy adsorption configurations corresponding to ~90° and ~70° rotations of the long axis of **ECG** with respect to the *a* lattice vector (Fig. S17a, b), with the respective adsorption energies being –9.51 eV (Fig. S17a) and –9.38 eV (Fig. S17b), corresponding to an interaction of 123–125 meV per carbon atom. Ring puckering of 0.55–0.60 Å was observed in both configurations, with the spin-polarized edges being closer to the surface than the center of the molecule, in agreement with the AFM data (Figs. 2 and S16).

To estimate charge transfer, we calculated the Hirschfeld atomic charges[11] at the geometry shown in Fig. S17a. At the PBE level of theory, Hirschfeld charges indicate ~1.16 electrons are transferred from the surface to **ECG**, suggesting an anionic ground state. However, the extent of charge transfer is likely overestimated at the PBE level of theory due to self-interaction error.[12] To obtain a more realistic measure, we also computed Hirschfeld charges at the PBE+U level of theory, applying a moderate Hubbard-like term U = 4 eV[13] to the carbon *p* orbitals. This reduced the charge transfer to ~0.71 electrons, indicating substantial charge transfer and ground state of **ECG** with anionic character. The negative charge is distributed across the carbon atoms at the rim of **ECG** (Fig. S18).

1.5. Multiconfigurational quantum chemistry calculations.

The solutions of a bilinear (BL) Heisenberg Hamiltonian between particles *i* and *j* coupled by J_{ij}

$$\hat{H}_{\text{BL}} = -\sum_{i,j} J_{ij}\, \mathbf{S}_i \cdot \mathbf{S}_j \quad (4)$$

for a system of four spin-1/2 particles are two singlets (S = 0), three triplets (S = 1), and one quintet (S = 2). The energies of these states were obtained from first principles by a difference-dedicated configuration interaction (DDCI) calculation based on a complete active space (CAS) calculation with four electrons and four orbitals in an active space on the gas-phase optimized **ECG** geometry. The minimal basis set (STO-3G; one p_z orbital per carbon) was used. DDCI was chosen due to its excellent performance in modeling exchange couplings.[14] We note that a previous study found that DDCI-determined superexchange-dominated exchange couplings increased in magnitude with the size of the basis set, so our use of a minimal basis set might slightly underestimate the value of J.[15]

The energy spectrum of magnetic excitations obtained using DDCI is shown in the first column of Fig. S20c. To determine exchange couplings, DDCI results were fit to the BL Hamiltonian (eq. 4), with results shown in Fig. S20a and the second column of Fig. S20c. As the (ferromagnetic) coupling between electrons at the same side of **ECG** is much stronger than the coupling between electrons on the opposite sides ($|J_1| >> |J_2| \approx |J_3|$; see Fig. S20a), the low-energy spectrum of **ECG** can also be described with the bilinear-biquadratic (BLBQ) Hamiltonian[16] applied to a two spin-1 particle system

$$\hat{H}_{\mathrm{BLBQ}} = \sum_{i,j} -J_{ij}\,\mathbf{S}_i \cdot \mathbf{S}_j - B_{ij}\left(\mathbf{S}_i \cdot \mathbf{S}_j\right)^2 \qquad (5)$$

Where J_{ij} is the bilinear and B_{ij} the biquadratic coupling between spin-1 particles i and j. Results of the BLBQ Hamiltonian are shown in Fig. S20b and the third column of Fig. S20c.

Effects of adsorption. The effect of adsorption of **ECG** on a metallic surface was approximately studied at the many-body level in two ways.

First, we estimated the screening effect of the surface on the obtained DDCI eigenvalues by employing a conductor-like polarizable continuum model (C-PCM)[17] with a high static dielectric constant (ε = 100) at the CASSCF(4,4) level of theory. Implicitly, this approach assumes no significant changes in density between CASSCF and CAS-DDCI wavefunctions, and it does not account for hybridization with the surface. While approximate, this approach shows essentially no changes in the energies of the low-lying magnetic excitations (Table S1).

Second, we recomputed the DDCI eigenvalues at the lowest-energy surface-adsorbed geometry of **ECG** obtained at the PBE-D3BJ level of theory (see Section 1.4 and Fig. S17a). The surface was not included in the calculations. This approach gave a reorganization energy of 0.75 eV, indicating strong interaction of **ECG** with the surface. At this geometry, the energies of low-lying magnetic excitations are slightly smaller (1.0–1.7 meV) than in the gas-phase geometry, while the energies of higher excitations are substantially altered (Table S1).

1.6. Simulation of AFM images.

AFM images of **ECG** were simulated by the probe-particle model implemented by Hapala et al.[18,19] The geometry of **ECG** corresponded to the one shown in Fig. S17a. The lateral and radial stiffness values of the carbon monoxide-functionalized tip were set to 0.5 and 30 N/m, respectively, with the oscillation amplitude set to 0.5 Å.

2. Supporting data

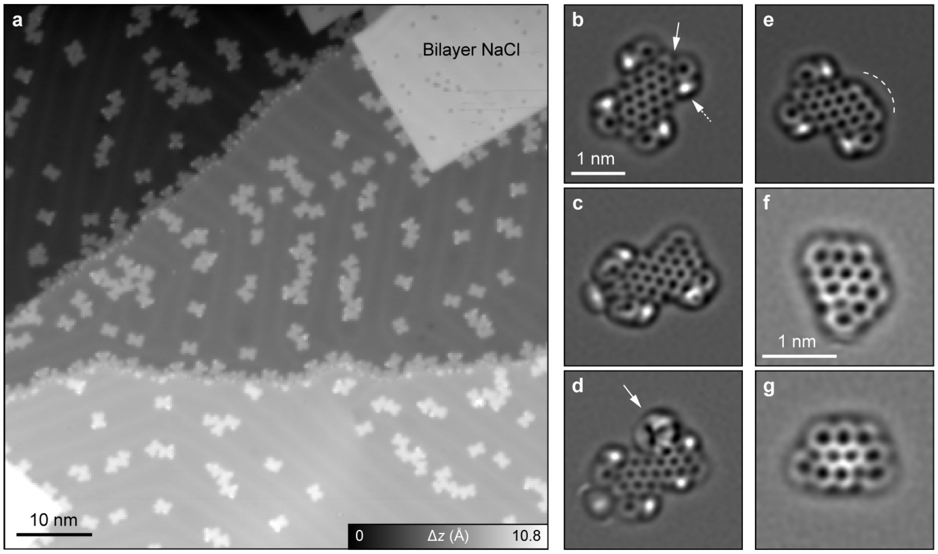

Figure S13. On-surface reactions of **1** on Au(111). (a) Overview STM image after annealing **1** on Au(111) at 600 K (*V* = 0.2 V, *I* = 1 pA). (b–g) Laplace-filtered AFM images of several isolated molecules on the surface. We always observed loss of one or more methyl groups in **1** that led to the formation of pentagonal rings upon cyclodehydrogenation reactions, indicated by the solid arrow in (b). Where methyl groups are not lost and hexagonal rings are formed, we mostly observed incomplete dehydrogenation leading to methylene moieties, indicated by the dashed arrow in (b). The molecules in (b), (c) and (d) result from loss of four, three and two methyl groups, respectively. The molecule in (d) additionally contains an unreacted xylyl group indicated by the arrow. The molecule in (e) exhibits loss of three methyl and one xylyl groups (the dashed curve indicates the region from where the xylyl group is lost). The molecules in (f) and (g) result from fragmentation of the precursor into 4,8-bis(2,6-dimethylphenyl)-4,8-dihydrodibenzo[*cd*,*mn*]pyrene units, along with the loss of one (f) and two (g) methyl groups. STM set-point for AFM images: *V* = 0.2 V, *I* = 1 pA on Au(111); Δ*z* = –1.5 (b–f) and –1.7 Å (g). The scale bar in (b) also applies to (c–e), and the scale bar in (f) also applies to (g).

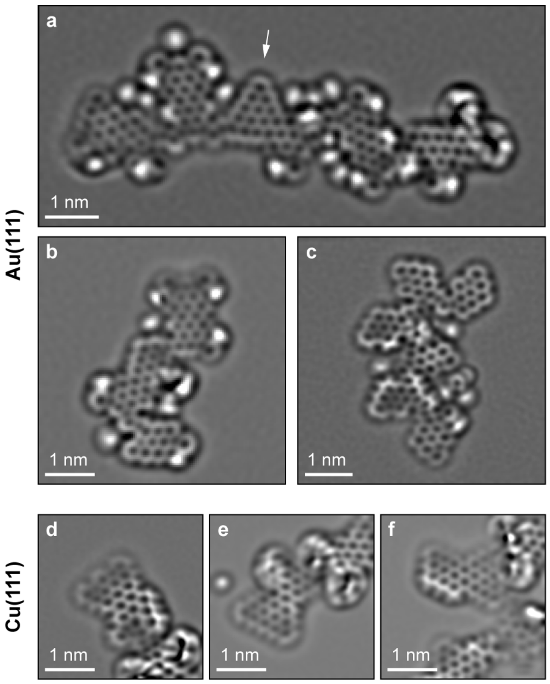

Figure S14. AFM images of molecular clusters on Au(111) (a–c) and Cu(111) (d–f), consisting of covalently-bonded molecules. The clusters were found to be larger on Au(111), which may be explained by the higher mobility of the molecules on Au(111) compared with Cu(111). The arrow in (a) indicates a rare instance of no loss of methyl or xylyl groups from one half of **1** on Au(111), leading to a π-extended olympicenyl[20] diradical motif upon cyclization reactions (see also Fig. S15d). STM set-points: V = 0.2 V, I = 1 pA on Au(111) and 0.5 pA on Cu(111); Δz = –1.5 (a), –1.3 (b), –1.8 (c) and –2.8 Å (d–f).

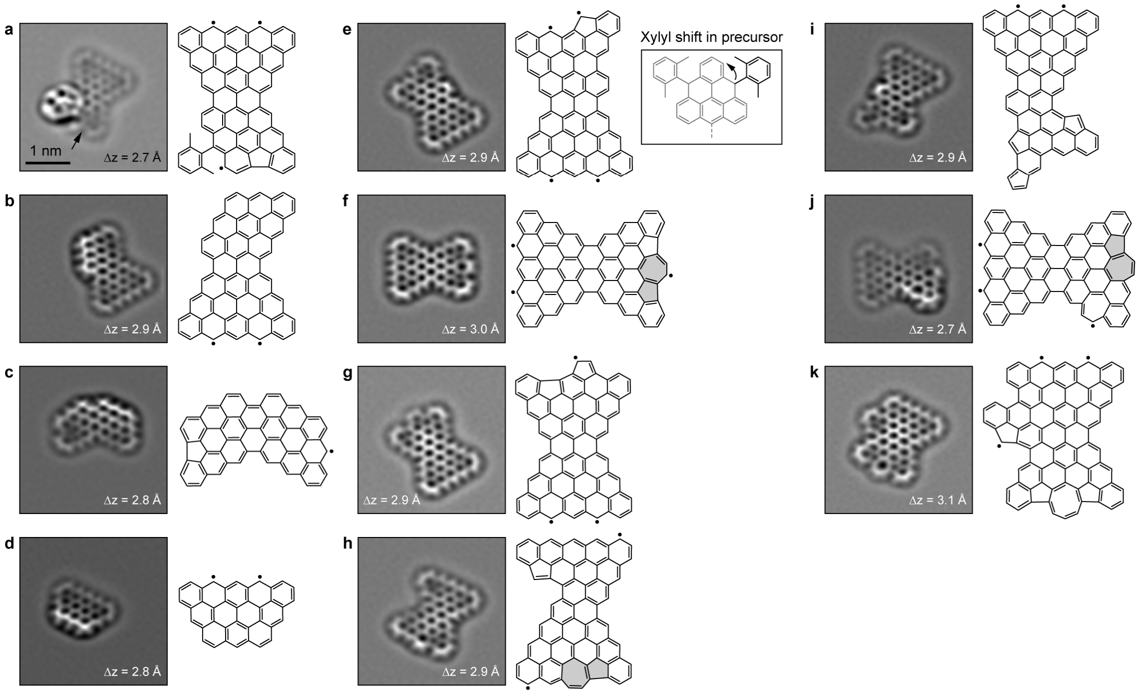

Figure S15. (a–k) Laplace-filtered AFM images and corresponding chemical structures of selected molecules that do not correspond to **ECG**, obtained after annealing **1** on Cu(111) at 530 K. The

molecules in (a–d) result from loss of methyl and/or xylyl groups (a–c), and precursor fragmentation (d), as also observed on Au(111) (Fig. S13). The molecule in (e) results from a xylyl shift in **1** and subsequent cyclization reactions. The molecules in (f–k) result from more complex chemical reactions. Some of them (g, j, k) contain more carbon atoms than in **ECG**, which could be explained by the reaction of molecular species with small fragments (such as methyl radicals). Other molecules contain azulene moieties (gray filled rings) that may result from ring rearrangement reactions. STM set-point: V = 0.2 V, I = 0.5 pA on Cu(111). The scale bar in (a) also applies to (b–k).

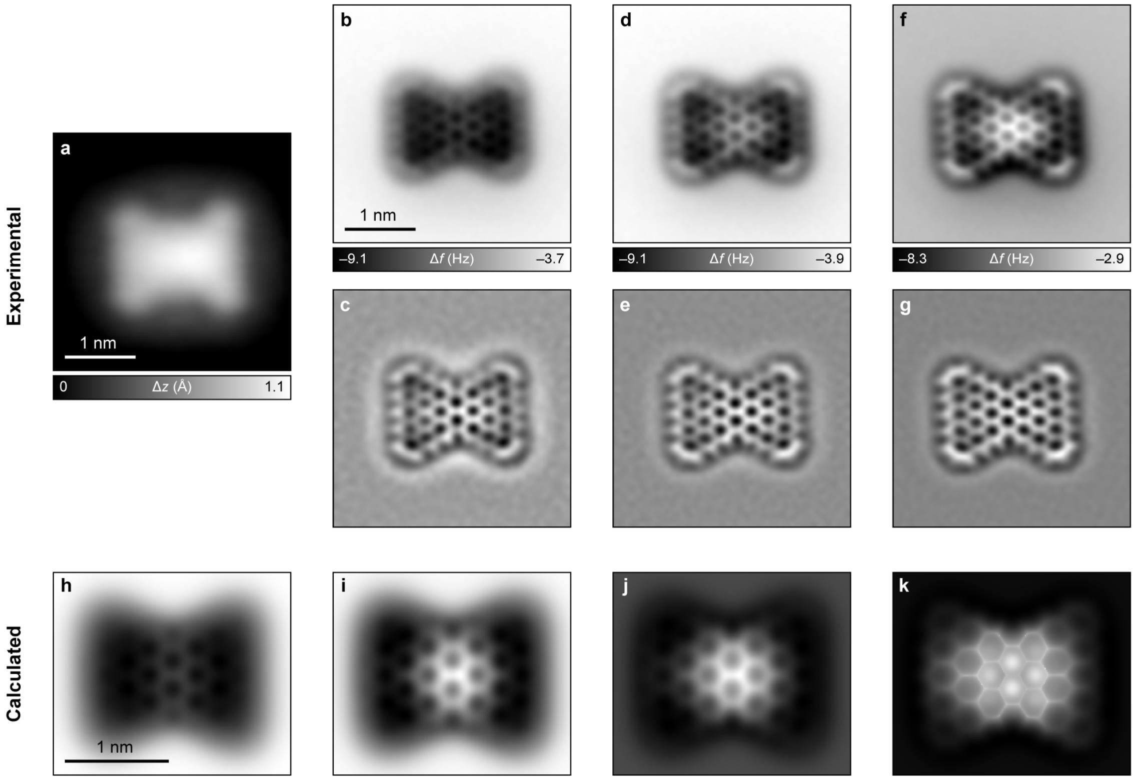

Figure S16. Height-dependent AFM imaging of **ECG** on Cu(111) and AFM simulations. (a) STM image of **ECG** on Cu(111) (V = 0.2 V, I = 0.5 pA). (b–g) AFM images (b, d, f) and corresponding Laplace-filtered images (c, e, g) of **ECG** acquired at different tip heights. STM set-point: V = 0.2 V, I = 0.5 pA on Cu(111); Δz = –2.6 (b, c), –2.8 (d, e) and –3.0 Å (f,g). (h–k) Simulated AFM images of **ECG** on Cu(111) at decreasing tip-molecule distances from left to right: 8.0 (h), 7.8 (i), 7.6 (j) and 7.2 Å (k). The scale bar in (b) also applies to (c–g), and the scale bar in (h) also applies to (i–k).

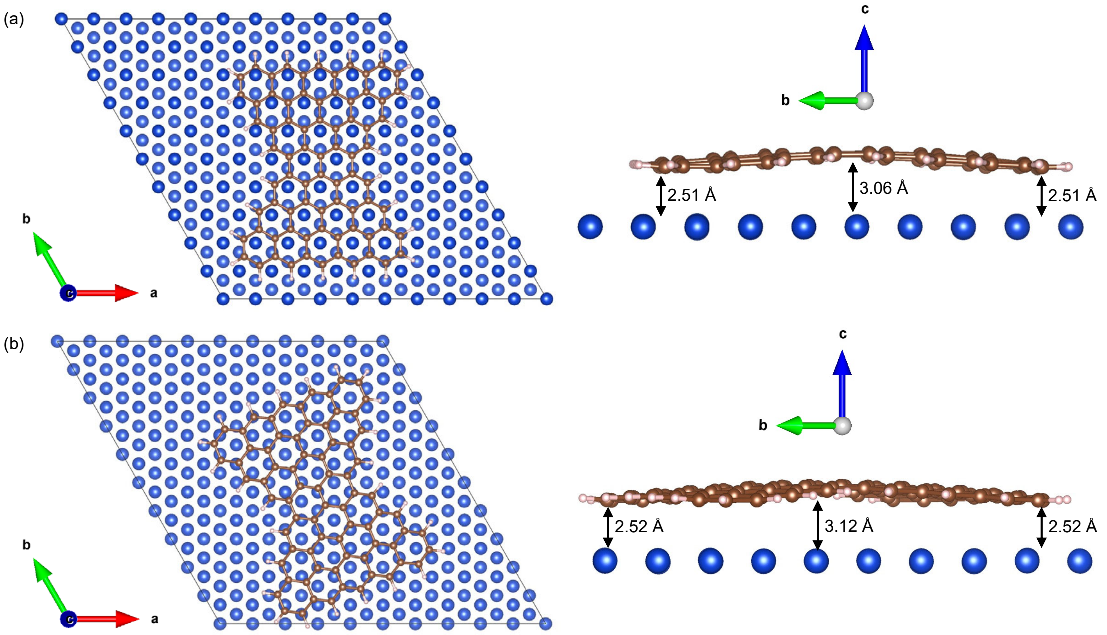

Figure S17. Top (left) and side (right) views of the DFT-optimized geometries of **ECG** on Cu(111). The two lowest-energy configurations are shown, with the long axis of **ECG** rotated by (a) ~90° with respect to lattice vector *a* and E_{ads} = –9.51 eV, and (b) ~70° with respect to *a* and E_{ads} = –9.38 eV.

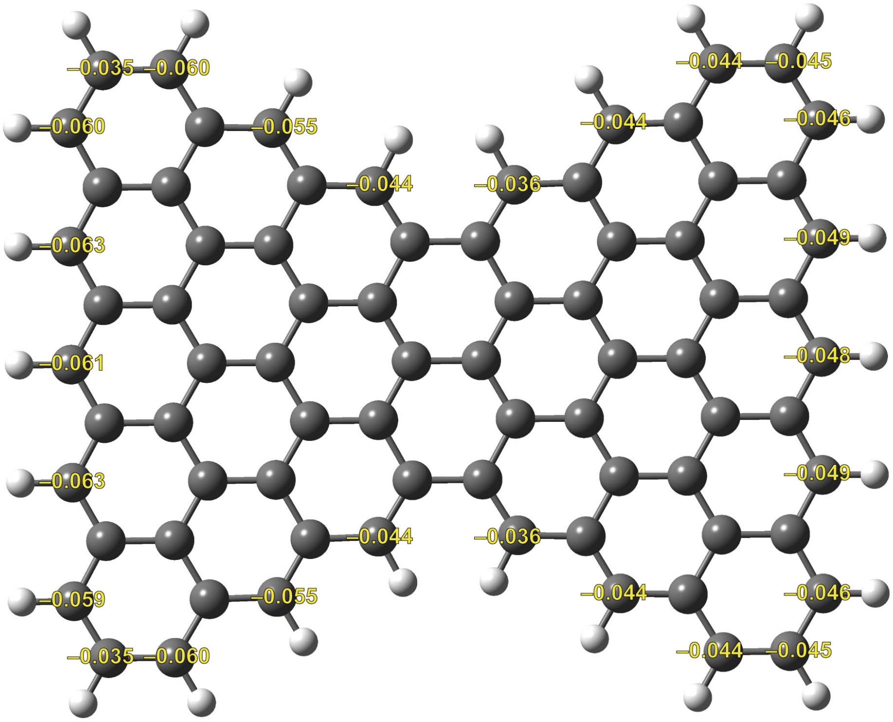

Figure S18. Hirschfeld charges in **ECG** on Cu(111) in the relaxed geometry shown in Fig. S17a at the PBE+*U* (*U* = 4 eV) level of theory. Only charges smaller than –0.02 *e* are shown.

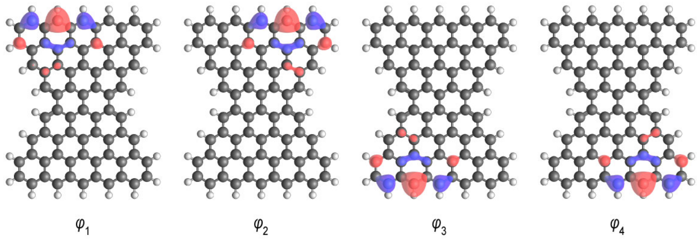

Figure S19. CASSCF(4,4) localized orbitals of **ECG**.

Note. Here, we provide the composition of the CASSCF(4,4)-DDCI many-body ground and excited states of **ECG** in the localized orbital basis $|\varphi_1\varphi_2\varphi_3\varphi_4\rangle$ (Fig. S19).

$$S_0 = 0.58(|\uparrow\uparrow\downarrow\downarrow\rangle + |\downarrow\downarrow\uparrow\uparrow\rangle) - 0.29(|\uparrow\downarrow\uparrow\downarrow\rangle + |\uparrow\downarrow\downarrow\uparrow\rangle + |\downarrow\uparrow\uparrow\downarrow\rangle + |\downarrow\uparrow\downarrow\uparrow\rangle)$$

$$T_1 = 0.5(|\downarrow\uparrow\uparrow\uparrow\rangle + |\uparrow\downarrow\uparrow\uparrow\rangle - |\uparrow\uparrow\downarrow\uparrow\rangle - |\uparrow\uparrow\uparrow\downarrow\rangle)$$

$$Q_1 = |\uparrow\uparrow\uparrow\uparrow\rangle$$

$$T_2 = 0.5(-|\downarrow\uparrow\uparrow\uparrow\rangle + |\uparrow\downarrow\uparrow\uparrow\rangle - |\uparrow\uparrow\downarrow\uparrow\rangle + |\uparrow\uparrow\uparrow\downarrow\rangle)$$

$$T_3 = 0.5(-|\downarrow\uparrow\uparrow\uparrow\rangle + |\uparrow\downarrow\uparrow\uparrow\rangle + |\uparrow\uparrow\downarrow\uparrow\rangle - |\uparrow\uparrow\uparrow\downarrow\rangle)$$

$$S_1 = 0.50(|\uparrow\downarrow\downarrow\uparrow\rangle + |\downarrow\uparrow\uparrow\downarrow\rangle - |\uparrow\downarrow\uparrow\downarrow\rangle - |\downarrow\uparrow\downarrow\uparrow\rangle)$$

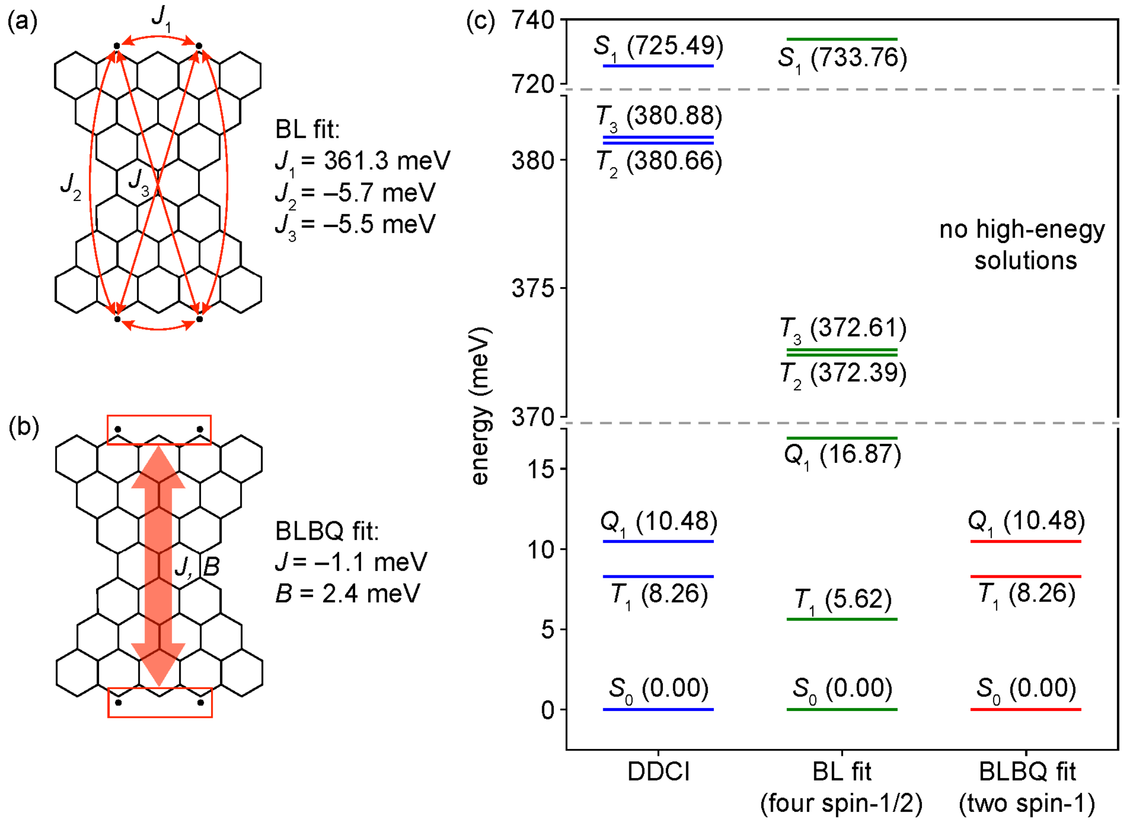

Figure S20. Magnetic interactions in **ECG**. (a) The spin-1/2 bilinear (BL) and (b) the spin-1 bilinear-biquadratic (BLBQ) effective Hamiltonians, with exchange couplings and their fit to DDCI results shown. (c) Energy spectrum obtained from DDCI (first column), and the fitted BL (second column) and BLBQ (third column) Hamiltonians.

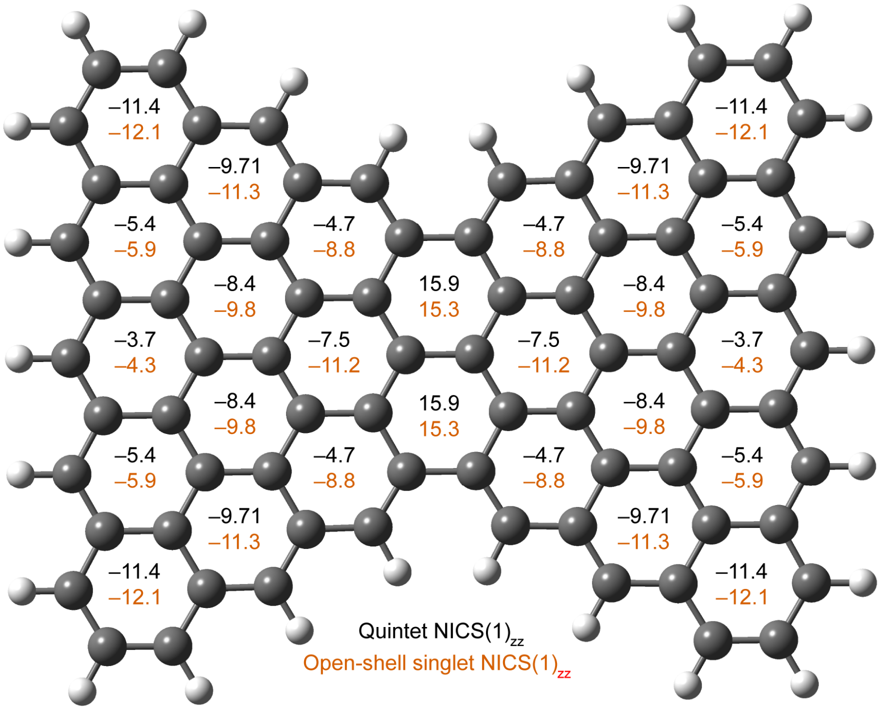

Figure S21. Comparison of DFT-calculated NICS(1)$_{zz}$ values at B3LYP/def2-TZVP in the quintet and open-shell singlet states. The open-shell singlet state was obtained by orbital rotation from the quintet wavefunction followed by stability analysis.

Table 1. Effects of surface adsorption on the magnetism of **ECG**. Energies are in meV (and indicated relative to the energy of the S_0 state for the second and third columns). Renormalization energy ΔE for each state is calculated using C-PCM (ε = 100) at the CASSCF(4,4) level.

State	DDCI (gas-phase geom.)	DDCI (surf. geom.)	ΔE, C-PCM (gas-phase geom.)	ΔE, C-PCM (surf. geom.)
T_1	8.26	6.57	0.01	0.01
Q_1	10.48	9.56	0.02	0.04
T_2	380.66	265.75	1.80	2.45
T_3	380.88	341.64	1.79	0.73
S_1	725.49	597.43	3.55	3.16

3. References

(1) Donovan, P. M.; Scott, L. T. 4,11-Bisanthenequinone and 10,10′-Bianthrone: Simple One-Step Syntheses from Anthrone. *Polycycl. Aromat. Compd.* **2008**, *28* (2), 128–135.

(2) Su, J.; Telychko, M.; Hu, P.; Macam, G.; Mutombo, P.; Zhang, H.; Bao, Y.; Cheng, F.; Huang, Z.-Q.; Qiu, Z.; Tan, S. J. R.; Lin, H.; Jelínek, P.; Chuang, F.-C.; Wu, J.; Lu, J. Atomically Precise Bottom-up Synthesis of π-Extended [5]Triangulene. *Sci. Adv.* **2019**, *5* (7), eaav7717.

(3) Giessibl, F. J. High-Speed Force Sensor for Force Microscopy and Profilometry Utilizing a Quartz Tuning Fork. *Appl. Phys. Lett.* **1998**, *73* (26), 3956–3958.

(4) Albrecht, T. R.; Grütter, P.; Horne, D.; Rugar, D. Frequency Modulation Detection Using High-Q Cantilevers for Enhanced Force Microscope Sensitivity. *J. Appl. Phys.* **1991**, *69* (2), 668–673.

(5) Stephens, P. J.; Devlin, F. J.; Chabalowski, C. F.; Frisch, M. J. Ab Initio Calculation of Vibrational Absorption and Circular Dichroism Spectra Using Density Functional Force Fields. *J. Phys. Chem.* **1994**, *98* (45), 11623–11627.

(6) Weigend, F.; Ahlrichs, R. Balanced Basis Sets of Split Valence, Triple Zeta Valence and Quadruple Zeta Valence Quality for H to Rn: Design and Assessment of Accuracy. *Phys. Chem. Chem. Phys.* **2005**, *7* (18), 3297–3305.

(7) Frisch, M. J.; Trucks, G. W.; Schlegel, H. B.; Scuseria, G. E.; Robb, M. A.; Cheeseman, J. R.; Scalmani, G.; Barone, V.; Petersson, G. A.; Nakatsuji, H.; Li, X.; Caricato, M.; Marenich, A. V.; Bloino, J.; Janesko, B. G.; Gomperts, R.; Mennucci, B.; Hratchian, H. P.; Ortiz, J. V.; Izmaylov, A. F.; Sonnenberg, J. L.; Williams-Young, D.; Ding, F.; Lipparini, F.; Egidi, F.; Goings, J.; Peng, B.; Petrone, A.; Henderson, T.; Ranasinghe, D.; Zakrzewski, V. G.; Gao, J.; Rega, N.; Zheng, G.; Liang, W.; Hada, M.; Ehara, M.; Toyota, K.; Fukuda, R.; Hasegawa, J.; Ishida, M.; Nakajima, T.; Honda, Y.; Kitao, O.; Nakai, H.; Vreven, T.; Throssell, K.; Montgomery, J. A., Jr.; Peralta, J. E.; Ogliaro, F.; Bearpark, M. J.; Heyd, J. J.; Brothers, E. N.; Kudin, K. N.; Staroverov, V. N.; Keith, T. A.; Kobayashi, R.; Normand, J.; Raghavachari, K.; Rendell, A. P.; Burant, J. C.; Iyengar, S. S.; Tomasi, J.; Cossi, M.; Millam, J. M.; Klene, M.; Adamo, C.; Cammi, R.; Ochterski, J. W.; Martin, R. L.; Morokuma, K.; Farkas, O.; Foresman, J. B.; Fox, D. J. *Gaussian 16, Revision C.01*; Gaussian, Inc.: Wallingford, CT, USA, 2016.

(8) Monaco, G.; Summa, F. F.; Zanasi, R. Program Package for the Calculation of Origin-Independent Electron Current Density and Derived Magnetic Properties in Molecular Systems. *J. Chem. Inf. Model.* **2021**, *61* (1), 270–283.

(9) Perdew, J. P.; Burke, K.; Ernzerhof, M. Generalized Gradient Approximation Made Simple. *Phys. Rev. Lett.* **1996**, *77* (18), 3865–3868.

(10) Grimme, S.; Ehrlich, S.; Goerigk, L. Effect of the Damping Function in Dispersion Corrected Density Functional Theory. *J. Comput. Chem.* **2011**, *32* (7), 1456–1465.

(11) Bultinck, P.; Van Alsenoy, C.; Ayers, P. W.; Carbó-Dorca, R. Critical Analysis and Extension of the Hirshfeld Atoms in Molecules. *J. Chem. Phys.* **2007**, *126* (14), 144111.

(12) Patra, A.; Peng, H.; Sun, J.; Perdew, J. P. Rethinking CO Adsorption on Transition-Metal Surfaces: Effect of Density-Driven Self-Interaction Errors. *Phys. Rev. B* **2019**, *100* (3), 035442.

(13) Jacob, D.; Ortiz, R.; Fernández-Rossier, J. Renormalization of Spin Excitations and Kondo Effect in Open-Shell Nanographenes. *Phys. Rev. B* **2021**, *104* (7), 075404.

(14) Malrieu, J. P.; Caballol, R.; Calzado, C. J.; de Graaf, C.; Guihéry, N. Magnetic Interactions in Molecules and Highly Correlated Materials: Physical Content, Analytical Derivation, and Rigorous Extraction of Magnetic Hamiltonians. *Chem. Rev.* **2014**, *114* (1), 429–492.

(15) Bogdanov, N. A.; Li Manni, G.; Sharma, S.; Gunnarsson, O.; Alavi, A. Enhancement of Superexchange Due to Synergetic Breathing and Hopping in Corner-Sharing Cuprates. *Nat. Phys.* **2022**, *18* (2), 190–195.

(16) Henriques, J. C. G.; Fernández-Rossier, J. Anatomy of Linear and Nonlinear Intermolecular Exchange in *S* = 1 Nanographene. *Phys. Rev. B* **2023**, *108* (15), 155423.

(17) Barone, V.; Cossi, M. Quantum Calculation of Molecular Energies and Energy Gradients in Solution by a Conductor Solvent Model. *J. Phys. Chem. A* **1998**, *102* (11), 1995–2001.

(18) Hapala, P.; Kichin, G.; Wagner, C.; Tauz, F. S.; Temirov, R.; Jelínek, P. Mechanism of high-resolution STM/AFM imaging with functionalized tips. *Phys. Rev. B* **2014**, *90* (8), 085421.

(19) PyProbe Web interface 0.2 Alpha http://ppr.fzu.cz/.

(20) Mistry, A.; Moreton, B.; Schuler, B.; Mohn, F.; Meyer, G.; Gross, L.; Williams, A.; Scott, P.; Costantini, G.; Fox, D. J. The Synthesis and STM/AFM Imaging of ‘Olympicene’ Benzo[*cd*]Pyrenes. *Chem. Eur. J.* **2015**, *21* (5), 2011–2018.
